# The Shortcomings of Plenum Ventilation

**Published:** 1906-05-05

**Authors:** 


### THE SHORTCOMINGS OF PLENUM VENTILATION.

The "Plenum" system of ventilation has for some years been the subject of one of the controversies that are so common in the medical and in the engineering world, and, in fact, wherever progress is the order of the day. The development of the system, like that of so many other things, has been very gradual. The men who have had the matter in hand, have felt their way, step by step, and they have not always made use of the knowledge that lay at their doors, and to be had for the asking. Ventilation has been carried on in coal mining for a great number of years, and while the arrangements are necessarily not the same, the principles are, and a study of mining practice would have saved a good deal of money and time in the early days of the development of the system. It would be of service even now to those who have work of the kind in hand. Want of knowledge of the kind led almost unavoidably to failure to effect the primary objects of the system. Draughts were produced instead of ventilation, and those who had designed the apparatus were surprised that it was so. But in the latest practice all this has been overcome. A gentle air current is produced that is in no sense a draught, and that is not to be distinguished by the senses from Nature's best and healthiest arrangements. The ventilating current is so efficient in the best practice that all smells are removed as soon as formed. There is no apparent change in the air when passing from a ward to the corridor, or *vice versa.* There is no smell of drugs, nor even of stale smoke, where male convalescents have been allowed to smoke in one of the day rooms. And the air is maintained at a uniform temperature day and night. Further, it is claimed by Mr. Henman, the architect of the Birmingham General Hospital, who has thoroughly mastered the principles of air currents, that smaller wards are possible with the system, because the air is so constantly changed, and that the decreased cost of the wards more than pays for the heavy cost of the ducts and apparatus required. It may be mentioned in passing that one cause of non-success of the early attempts to produce the gentle air currents required, and the large power that some of them take to move the air, is due to the ducts not being large enough. Small ducts mean large power and a high velocity in the air, the one meaning expense and the other draughts.

But after all credit has been given for the above, there is still something wanting. It is difficult to find a doctor who is heart and soul in favour of the system. Doctors and nurses object to it in their quarters, and in the hospital at Belfast, which was designed by Mr. Henman after the completion of the hospital at Birmingham, the residential quarters were expressly excluded. The expression one commonly hears from men who have studied the question—doctors, physicists, and others—is, "There appears to be something taken out of the air." And it appears to the present writer that the feeling is correct that something is taken out—namely, a portion of the oxygen. Doctors and nurses in residence complain of a feeling of staleness after a night's rest in a Plenum atmosphere, or after sitting in it for some time. Doctors complain that convalescents do not get on as quickly as they should. They reach a certain stage fairly well, but afterwards progress very slowly unless they are removed from the hospital. And there are other instances. The explanation the writer ventures to offer is the temperature being maintained for a large portion of the year, and particularly at night, at a higher figure than would rule naturally, the proportion of oxygen present is smaller. An increase of the temperature of a room causes an expansion of the air in the room, and some of that which was in the room at the lower temperature escapes if free to do so. That which is left in the room is thinner, so to speak, poorer in oxygen. It has less oxygen by weight. And this applies equally to air supplied at a higher temperature. It is also thinner, weaker in oxygen. The difference is usually not great, but it is operating the whole time, and it will probably be the experience of every medical man, as it is of every engineer, that it is very often the small things, continually in operation, that are so powerful for good or ill. A difference of temperature of 5 degrees Fahrenheit, makes approximately a difference of 1 per cent. in the oxygen present in any volume of air, leaving out for the moment the question of the moisture. The bracing climate of Switzerland, of Canada, and of the seashore, are due to the presence of increased quantities of oxygen, or ozone, but the quantities are not large. The moisture has also a very important effect. Any moisture in the air is present at the expense of a certain quantity of the air itself, so that the greater the percentage of moisture the smaller the quantity of oxygen available. It is not usual, in the writer's experience, to control the humidity of each ward or room separately, and it is therefore probable that some parts of the building will have less oxygen than they otherwise would have owing to the presence of a larger percentage of moisture than it requires. It would probably be expensive and difficult to efficiently control the percentage of moisture to different parts of a building supplied by one fan; and on the other hand moisture plays strange tricks. Higher temperatures enable air to absorb larger quantities, which the advent of a lowered temperature, or changes in other physical conditions, oblige it to deposit, re-evaporation again taking place, and so on.

There is one other point, the effect of the adoption of the Plenum system by a number of buildings closely packed together in a town upon the atmosphere. Where the building is in the country, away from others, no harm is done; but where all the buildings are drawing from a common reservoir of air, into which they are delivering polluted air, the matter may become so serious as to neutralise any good effects obtainable from the system.

